# Supplementary material for: Hedera rhombea inhibits the biofilm formation of Candida, thereby increases the susceptibility to antifungal agent, and reduces infection
Source: PLoS One. 2021 Oct 6;16(10):e0258108. doi: 10.1371/journal.pone.0258108 (PMC8494327; doi:10.1371/journal.pone.0258108)
Supplement: Attachment — Submitted filename: Rebuttal_PLOS One_0511_Final.docx [file pone.0258108.s002.docx]

*Hedera rhombea* inhibits the biofilm formation of *Candida*, thereby increases the susceptibility of antifungal agent, and reduces infection.

We thank the editors and the reviewers for their thoughtful and helpful comments. We have addressed, in a point-by-point manner, all the suggestions and queries from the journal and the reviewer, and marked with red in manuscript. The input from the reviewers has allowed us to improve the clarity and quality of our paper. We have included below our point-by-point response to the reviewers’ comments and have included these additions and alterations to the revised manuscript.

**Journal Requirements:**

**When submitting your revision, we need you to address these additional requirements.**

**1. Please ensure that your manuscript meets PLOS ONE's style requirements, including those for file naming. The PLOS ONE style templates can be found at**

https://journals.plos.org/plosone/s/file?id=wjVg/PLOSOne_formatting_sample_main_body.pdf and

https://journals.plos.org/plosone/s/file?id=ba62/PLOSOne_formatting_sample_title_authors_affiliations.pdf

: Thanks a lot for your approval and professional advice on the manuscript. We check manuscript again for PLOS ONE's style requirements.

**2. Please include a separate caption for each figure in your manuscript.**

: Thanks for your suggestion. That part has been modified.

**3. We noticed you have some minor occurrence of overlapping text with the following previous publication(s), which needs to be addressed:**

https://journals.plos.org/plosone/article?id=10.1371/journal.pone.0167470

: Thank you very much. We checked and modified manuscript again compare with that reference.

**In your revision ensure you cite all your sources (including your own works), and quote or rephrase any duplicated text outside the methods section. Further consideration is dependent on these concerns being addressed.**

: Thanks for the comments. We checked manuscript again concerned about the citation.

[Note: HTML markup is below. Please do not edit.]

Reviewers' comments:

Reviewer's Responses to Questions

Comments to the Author

1. Is the manuscript technically sound, and do the data support the conclusions?

The manuscript must describe a technically sound piece of scientific research with data that supports the conclusions. Experiments must have been conducted rigorously, with appropriate controls, replication, and sample sizes. The conclusions must be drawn appropriately based on the data presented.

Reviewer #1: Partly

Reviewer #2: Partly

2. Has the statistical analysis been performed appropriately and rigorously?

Reviewer #1: Yes

Reviewer #2: No

Thank you very much. We checked the results whether the error bar and statistically appropriately presented again.

3. Have the authors made all data underlying the findings in their manuscript fully available?

The PLOS Data policy requires authors to make all data underlying the findings described in their manuscript fully available without restriction, with rare exception (please refer to the Data Availability Statement in the manuscript PDF file). The data should be provided as part of the manuscript or its supporting information, or deposited to a public repository. For example, in addition to summary statistics, the data points behind means, medians and variance measures should be available. If there are restrictions on publicly sharing data—e.g. participant privacy or use of data from a third party—those must be specified.

Reviewer #1: Yes

Reviewer #2: Yes

4. Is the manuscript presented in an intelligible fashion and written in standard English?

PLOS ONE does not copyedit accepted manuscripts, so the language in submitted articles must be clear, correct, and unambiguous. Any typographical or grammatical errors should be corrected at revision, so please note any specific errors here.

Reviewer #1: Yes

Reviewer #2: No

Thank you very much. We checked the typing and grammatical errors again.

5. Review Comments to the Author

Please use the space provided to explain your answers to the questions above. You may also include additional comments for the author, including concerns about dual publication, research ethics, or publication ethics. (Please upload your review as an attachment if it exceeds 20,000 characters)

**Reviewer #1: Language and Format**

**A native English speaker should be consulted to address language issues. The errors are generally minor, but they are found throughout the paper. For instance, the first sentence of the introduction should read "...with impaired response is able to persist...and often leads to..."**

: Thanks for your feedback on this, and we have changed what we wrote in the introduction based on the recommendations.

*Candida* *albicans* is an opportunistic pathogen which is responsible for systemic infections in immunocompromised patients. *C.* *albicans* can persist inside the host due to the drug resistance traits, and often lead to the failure of therapeutic strategies [1].

**In Candida, gene names are italicized.**

Thank you for your suggestion. we changed all the style of gene names based on the recommendation.

: To know the molecular mechanisms of biofilm formation inhibition by extract, qRT-PCR analysis was done and found dose-dependently reduced the expression of hyphal-associated genes (*ALS3, ECE1, HWP1,* *PGA50* and *PBR1*), extracellular matrix genes (*GSC1, ZAP1, ADH5* and *CSH1*), Ras1-cAMP-PKA pathway genes (*CYR1, EFG1* and *RAS1*), Cph2-Tec1 pathway gene (*TEC1*) and MAP kinases pathway gene (*HST7*).

**Title**

**As noted below, virulence is not tested in this paper.**

: Thank you for your suggestion. we though that infection and adherence are belonging to the virulence but based on the reviewer comments, we changed to the infection rather than virulence, that is more reasonable.

*Hedera rhombea* inhibits the biofilm formation of *Candida*, thereby increases the susceptibility of antifungal agent, and reduces infection.

**Abstract**

**Reference is made to biofilm cellular density, but there are no results regarding this in the paper.**

**There is reference to bacterial growth.**

: Thank you very much. we have made corrections to what you said and added references to references section.

**Introduction**

**What else is known about extracts from this plant?**

**Why is there no reference to the following paper, in particular the impact on growth: Choi, H.A.; Cheong, D.E.; Lim, H.D.; Kim, W.H.; Ham, M.H.; Oh, M.H.; Wu, Y.; Shin, H.J.; Kim, G.J. Antimicrobial and anti-biofilm activities of the methanol extracts of medicinal plants against dental pathogens Streptococcus mutans and Candida albicans. J. Microbiol. Biotechnol. 2017, 27, 1242–1248, doi:10.4014/jmb.1701.01026.**

: Thanks for your careful work. we added more of biological effects of *Hedera rhombea.*

In oriental medicine, *H. rhombea* is mainly used for arthritis, low back pain, hepatitis, high blood pressure, hemostasis, anti-rheumatism, facial paralysis, jaundice anti-inflammatory action, hypertension, and antitumor [27, 28, 29].

**Materials and Methods**

**There are a number of non-standard methods and incomplete descriptions.**

**In biofilm formation, the inoculum concentration is half standard an no reference is made to inoculum volume. Hypha inducing medium (YPD+serum) is used but is incubated at yeast temperature (30C). Was the destaining solution transferred to a fresh well for reading the OD?**

: Thanks a lot for your approval and professional advice on the manuscript. we changed the method appropriately.

*H. rhombea* extract ranging from 6.25 to 100μg/mL were prepared in 96-well flat-bottomed plates (SPL, Korea). Controls without test compounds were served. 1 × 10^6^ CFU/mL of *C. albicans* suspension were prepared in RPMI 1640 medium [26, 31, 32, 33, 34, 36, 37]. Then 100μl of the solution was inoculated into 96-well flat-bottomed plates. After incubation at 37°C for 24 h, washing out none adhere cells with PBS and 100μl of 1% aqueous crystal violet solution was reacted for 30min. Each well was washed three times with PBS and instantly de-stained with 150μl of 30% acetic acid for 15min. The absorbance was measured at 595nm with a microplate reader (Bio Tek Instruments, Korea). The experiments were performed in triplicate (Fig 1).

**In the qPCR description, what was the time of the culture with the compound? Was the incubation with the compound done shaking or was this static? How was the cDNA synthesis primed? How were the results normalized?**

: Thanks for your suggestion. we incubated *C. albicans* for 24 h with shaking and we changed the sentence including that.

*C. albicans* was grown overnight in RPMI 1640 and diluted to 1 × 10^6^ cells/mL. The diluted suspension with 6.25-100µg/mL of *H. rhombea* extract was incubated at 37℃ for 24 h with shaking. Total RNA was isolated using TRIzol reagent (Life Technology, Thermo Fisher Scientific, USA) according to the manufacturer’s instruction and the reverse transcriptase (NanoHelix, Korea) reaction was prepared using 1 µg of RNA to obtain cDNA.

qRT-PCR was carried out with the 2X SybrGreen qPCR Mater Mix (CellSafe, Korea). The transcript level of detected genes was calculated using the formula 2-^ΔΔCT^. Primer sequences used are listed in Table 2. *ACT1* was used as internal control [26, 30, 31, 32, 33, 34]. The experiments were performed in triplicate.

**In the time kill assay, there is a typo (fatal for fetal). Growth is described as being in the YPD and YPD+serum, but the graph has only one base medium.**

: Thank you for your careful work. we have done with YPD and YPD+serum, but result is from YPD. So, we changed the methods that the result obtained.

Fungal culture was prepared with the fresh YPD to 1 × 10^6^ cells/mL of *C. albicans* [30, 31, 32, 33, 38]. 100μg/mL of *H. rhombea* extract was added and then incubated at 37℃. The growth was evaluated by measuring OD_600_ using microplate reader after 0, 1, 2, 4, 8, 12, and 24 h [30, 31, 32]. The experiments were performed in triplicate.

**Results.**

**It would be helpful if the species name was included at the top of each graph so that the reader didn’t need to keep consulting the figure legend.**

: Thank you very much. we added the strain name on the individual figure.

Fig 1.

A

***C. albicans***

% of Biofilm formation

| H. rhombea extracts [μg/mL] | 송악 |
| --- | --- |
| 0 | 100 |
| 6.25 | 68.2781 |
| 12.5 | 50.5563 |
| 25 | 36.5033 |
| 50 | 35.298 |
| 100 | 31.1788 |

B

***C. tropicalis var. tropicalis***

% of Biofilm formation

| H. rhombea extracts [μg/mL] | 송악 |
| --- | --- |
| 0 | 100 |
| 6.25 | 71.8223 |
| 12.5 | 62.1923 |
| 25 | 57.9811 |
| 50 | 55.2872 |
| 100 | 43.0562 |

C

***C. parapsilosis var. parapsilosis***

% of Biofilm formation

| H. rhombea extracts [μg/mL] | 송악 |
| --- | --- |
| 0 | 100 |
| 6.25 | 47.1395 |
| 12.5 | 45.9531 |
| 25 | 38.3865 |
| 50 | 32.5073 |
| 100 | 25.2834 |

D

***C. glabrata***

% of Biofilm formation

| H. rhombea extracts [μg/mL] | 송악 |
| --- | --- |
| 0 | 100 |
| 6.25 | 51.6824 |
| 12.5 | 40.6923 |
| 25 | 39.8935 |
| 50 | 34.7374 |
| 100 | 33.8417 |

E

***C. tropicalis***

% of Biofilm formation

| H. rhombea extracts [μg/mL] | 송악 |
| --- | --- |
| 0 | 100 |
| 6.25 | 69.4731 |
| 12.5 | 52.8197 |
| 25 | 51.8136 |
| 50 | 47.7098 |
| 100 | 47.2333 |

F

***C. parapsilosis***

% of biofilm foramtion

| H. rhombea extracts [μg/mL] | 송악 |
| --- | --- |
| 0 | 100 |
| 6.25 | 63.5638 |
| 12.5 | 46.0889 |
| 25 | 40.4881 |
| 50 | 36.7491 |
| 100 | 33.9643 |

**In figure 3, it’s not clear if the graphed results are from one replicate or the sum total of all results. There is no n value for the number of cells examined. Why is the cell density so much lower in panel subpanel D of panels B and D than in the other panels? In spite of figure 7, this makes it look like the compound is affecting viability, not just hypha formation. Was the “0” sample without any additive, or with same solvent as the extract?**

: Thank you for your suggestion. First, in the case of the graph, the number of cells was counted by taking pictures of various directions. The number of cells was counted to at least 1,000, and the photographs taken under the microscope appear to have been taken so that the number of cells appears to be small.

**Dimorphic transition of *C. albicans***

*C. albicans* were grown overnight in YPD medium. 1 × 10^6^ cells/mL of *Candida* with or without extracts were incubated in RPMI 1640 medium and YPD media supplemented with 10% fatal bovine serum medium to induce dimorphic transition at 37°C for 4 h. Inhibition quantification of the yeast-to-hyphal-form transition was accomplished by counting the number of hyphae cells in the population as previously described [26, 32, 33, 34, 35, 36, 37]. More than 1,000 cells were counted for each well in duplicate, and all assays were repeated five times. Large sprout cells (i.e., sprouts are similar in size to the parent cell) counted as 2 cells, and small sprout cells (i.e., sprouts are smaller than the parent cell) counted as one cell. The hyphae, which is usually identified as consisting of several distinct hyphae cells, is counted as a single cell. This method, which was necessary because the hyphae-specific identification cells could not be quantified, also resulted in an under expression of the number of mycelial cells in the population. The reported percentage of hyphae is normalized to the percentage of probability hyphae when a numerator is not added. Since the number of cells observed in D is small, it seems to be the part where the number of observed cells is small. In the case of 0, the same solvent was added. Representative results of images were obtained using a fluorescence microscope (EVOS® FL, ThermoFisher Scientific, Waltham, MA, USA). The experiments were performed in triplicate.

**In figure 4, how many cells were counted?**

**The times in the graph for Figure 7 do not match those in the methods.**

: Thank you for your comments, this method is incubated for 4 h. More than 1,000 cells were counted for each well in duplicate, and all assays were repeated three times.

**Discussion.**

**There is a statement that various plant extracts “may not always do their work”. What is that supposed to mean? Figures 3 and 4 do not assess virulence of C. albicans although they examine filamentation and adhesion which are important virulence trait. There are a number of examples of C. albicans strains with adhesion defects which retain high virulence, so this statement needs to be modified.**

: Thank you for your careful work. We removed the sentence because it was an accident because the sentence did not clearly give meaning. Based on the reviewer's comments, we made all the toxic changes in this manuscript.

**Reviewer #2: This paper describes the influence of plant extract from Hederea rhombea on** **Candida albicans. The paper suggest a wide ranging influence of the plant extract making this a paper with potentially high impact.**

**There are issues throughout the paper with the English which start with the title. Virulence should not be used as it is not assessed. It should probably read something like "Hedera rhombea inhibits the biofilm formation of Candida and increases the susceptibility of the antifungal agent Miconazole". The issues continue throughout the paper and this really should be improved before further review.**

: Thank you for your suggestion. We checked manuscript again.

**The paper also doesn't address competing data from (https://doi.org/10.4014/jmb.1701.01026 J. Microbiol. Biotechnol. (2017), 27(7), 1242–1248) which showed less influence from Hederea rhombea. This should be referenced and discussed.**

: Thank you very much. we added more of biological effects of *Hedera rhombea.*

*Hedera rhombea* has traditionally been used for anti-rheumatism, facial paralysis, hepatitis, jaundice anti-inflammatory action, hypertension, and antitumor. This article refers to this paper for additional information on *Hedera rhombea.*

**The are issues with methods where they are non-standard and not fully described. The controls are either inappropriate or not fully described and this should be remedied. The biofilm data needs error bars and statistical analysis where significant difference is inferred. The protocols for biofilm formation are missing details...like how was the culture prepared prior to preparing cells for biofilm formation. How were the cells prepared for biofilm formation post initial growth. Non-standard film growth conditions are specified but not really justified/explained why 30 not 37 celsius? Why the prolonged incubation with crystal violet. Are they really scoring the actual biofilm and not the destain?. For the dimorphic assay... which RPMI was used how was it made/supplemented. How was it prepared to induce filamentation. The images also need to be better. Without this it is hard to evaluate these experiments. A better label could also be used for graph axis labels than H. rhombea. Perhaps use an acronym for your extract or just put extract.**

: Thank you for your careful review. In the error bar and statistical part of the biofilm data, the error bar was not visible as the graph it was reduced. Before preparing cells for biofilm formation, experiments were conducted using RPMI 1640 media, which induces biofilm after growing in YPD media first. When forming a biofilm, it was conducted at 37 degrees, not at 30 degrees, so it was incorrectly indicated in this paper. In the case of crystal violet, it was not cultured but was used to dye the biofilm, and it is used when dyeing the biofilm. For heteromorphic analysis, RPMI 1640 written in the reference was used. In the induction part of the filament, it was carried out according to the method in the reference literature. In the case of the extract, an abbreviation was used.

**Below are line by line suggestions that should improve the document. I would also suggest having some very fluent in English review the document.**

**Abstract suggestions:**

**change the first line to " Candida is an opportunistic pathogen and common cause of fungal infections worldwide. Anti-fungal use against Candida infections has resulted in the appearance of resistant strains. The limited choice of anti-fungal therapy means alternative strategies are needed to control fungal infectious diseases."**

: Thanks for your constructive suggestions. we modified it as you said.

*Candida* is an opportunistic pathogen and common cause of fungal infections worldwide. Anti-fungal use against Candida infections has resulted in the appearance of resistant strains. The limited choice of anti-fungal therapy means alternative strategies are needed to control fungal infectious diseases.

**The inhibition of biofilm.....(from line 6) should read something like "Biofilm formation was assessed using the crystal violet assay which showed a dose dependent reduction in the presence of extract**"

: Thank you for your careful work. we modified it as you said.

Biofilm formation was assessed using the crystal violet assay which showed a dose dependent reduction in the presence of extract with the biofilm formation inhibitory concentration of *C. albicans* (IC_50_ = 12.5μg/ml*), C. tropicalis var. tropicalis* (IC_50_ = 25μg/ml), *C. parapsilosis var. parapsilosis* (IC_50_ = 6.25$\mu$g/ml), *C. glabrata* (IC_50_ = 6.25$\mu$g/ml), *C. tropicalis* (IC_50_ = 12.5$\mu$g/ml), and *C. parapsilosis* (IC_50_ = 12.5$\mu$g/ml) without directly reducing *Candida* growth.

**Genes names should be in italics.**

: Thanks for your constructive suggestions. we modified it as you said.

To know the molecular mechanisms of biofilm formation inhibition by extract, qRT-PCR analysis was done and found dose-dependently reduced the expression of hyphal-associated genes (*ALS3, ECE1, HWP1, PGA50* and *PBR1*), extracellular matrix genes (*GSC1, ZAP1, ADH5* and *CSH1*), Ras1-cAMP-PKA pathway genes (*CYR1, EFG1* and *RAS1*), Cph2-Tec1 pathway gene (*TEC1*) and MAP kinases pathway gene (*HST7*).

**Introduction.**

**line 1 should read "....pathogen that is responsible for systemic infections in immunocompromised patients....."**

: Thank you for your careful work. we modified it as you said.

*Candida* *albicans* is an opportunistic pathogen which is responsible for systemic infections in immunocompromised patients. *C.* *albicans* can persist inside the host due to the drug resistance traits, and often lead to the failure of therapeutic strategies [1].

**line 10 should read "....most antifungal agents, ....."**

: Thank you for your suggestion. we modified it as you said.

In fact, adherent *C. albicans* cells without specific drug resistance genes are up to 1,000 times more resistant to common antifungal agents than planktonic cells [19]

**Line 12. Replace "Thereby" with Therefore.**

: Thank you very much. we modified it as you said.

Therefore, the biofilm of *C. albican* is a reservoir of viable fungal cells that can potentially cause systemic infections, with a mortality rate of around 40-60% [20, 21].

**Line 14 either delete Increasing or change to Increasingly**

: Thank you for your suggestion. we modified it as you said.

Efforts are being made to develop alternative strategies to eradicate biofilm-related infections [22, 23].

**Line 16 change to "Medicinal plants are used for diverse traditional methods....". end the sentence with "many other diseases"**

: Thank you very much. we modified it as you said.

Medicinal plants are used for diverse traditional methods to treat cancer, infection, fever, asthma, and many other diseases.

**Line 23 change to "....several fungi..."**

: Thank you for comments, we modified it as you said.

In this study, *H. rhombea* extract showed anti-biofilm formation activity against several fungi including *C. albicans, C. tropicalis, C. glabrata,* and *C. parapsilosis*.

**Line 24 change to "Particularly, anti-biofilm activity....."**

: Thank you very much. we modified it as you said.

Particularly, anti-biofilm activity increased susceptibility to antibiotics and reducing fungal infection. These results suggest that *H. rhombea* extract can be used as a potential anti-fungal adjuvant to control the biofilm-related infection.

**The methods need more detail. Please see above.**

**Discussion.**

**Line 3. Change to "Antifungal drugs can cause side effects and resistance and there have been multiple recent reports of resistance including in the emerging problematic organism Candida auris.**

: Thank you for your suggestion. we modified it as you said.

Many fungi including *Candida* live in and on the human body, but when *Candida* begins to grow uncontrollably, it can cause an infection known as candidiasis. In fact, *Candida* is the most common cause of fungal infections in humans [38]. Antifungal drugs can cause side effects and resistance, and there have been multiple recent reports of resistance including in the emerging problematic organism *Candida auris* [39, 40].

**At the bottom of the first paragraph. rephrase "those may not always do their work" to be more explicit about what their problem is.**

: Thank you very much. we modified it as you said.

**In the third paragraph (and later) ".....further studies must be conducted..." not followed!**

: Thank you for your suggestion. we modified it as you said.

Even though the mechanism of *Candida* biofilm formation is diverse depending on the species, *H. rhombea* extract inhibited *Candida* biofilm formation that suggests there should be a common mechanism to induce the biofilm among the *Candida* species, but further studies must be followed.

**Line 18 "... biofilm formation is related to the susceptibility..."**

: Thank you for your suggestion. we modified it as you said.

Moreover, *H. rhombea* increased the antifungal activity of miconazole, magnoflorine, and dioscin (Fig 2), and reduced *Candida* virulence (Fig 3 and 4), these results confirmed that biofilm formation is related to the susceptibility of antifungal agents and fungal virulence.

**Line 20 "....cell adhesion was significantly reduced by treatment with H. rhombea extract"**

: Thank you for your careful review. we modified it as you said.

Based on the gene expression analysis, the expression of genes related to biofilm formation, hyphae growth, and cell adhesion was significantly reduced by treatment with *H. rhombea* extract.

**on the following/last page**

**line 3 ".... and in vivo testing must be carried out."**

: Thank you very much. we modified it as you said.

So further studies including the precise target determination of *H. rhombea* extract, effector components and in vivo testing must be carried out.

**Line 7 is adjuvant really the right word. Perhaps simply say treatment option.**

: Thank you for your suggestion. we modified it as you said.

In conclusion, *H. rhombea* extract inhibited *C. albicans* biofilm formation, increased the antifungal activity of antibiotics and putative antifungal agents, and decreased fungal adherence to the host cell. Therefore, *H. rhombea* extract should be a good treatment option for biofilm-forming fungal infections.
