# Supplementary material for: Hedera rhombea inhibits the biofilm formation of Candida, thereby increases the susceptibility to antifungal agent, and reduces infection
Source: PLoS One. 2021 Oct 6;16(10):e0258108. doi: 10.1371/journal.pone.0258108 (PMC8494327; doi:10.1371/journal.pone.0258108)
Supplement: Attachment — Submitted filename: Rebuttal 2 07.28.docx [file pone.0258108.s003.docx]

*Hedera rhombea* inhibits the biofilm formation of *Candida*, thereby increases the susceptibility to antifungal agent, and reduces infection.

We thank the editors and the reviewers for their thoughtful and helpful comments. We have addressed, in a point-by-point manner, all the suggestions and queries from the journal and the reviewer and marked with red in manuscript. The input from the reviewers has allowed us to improve the clarity and quality of our paper. We have included below our point-by-point response to the reviewers’ comments and have included these additions and alterations to the revised manuscript.

**Reviewers' comments:**

**Reviewer's Responses to Questions**

**Comments to the Author**

**1. If the authors have adequately addressed your comments raised in a previous round of review and you feel that this manuscript is now acceptable for publication, you may indicate that here to bypass the “Comments to the Author” section, enter your conflict of interest statement in the “Confidential to Editor” section, and submit your "Accept" recommendation.**

Reviewer #1: (No Response)

Reviewer #2: (No Response)

**2. Is the manuscript technically sound, and do the data support the conclusions?**

**The manuscript must describe a technically sound piece of scientific research with data that supports the conclusions. Experiments must have been conducted rigorously, with appropriate controls, replication, and sample sizes. The conclusions must be drawn appropriately based on the data presented.**

Reviewer #1: Partly

Reviewer #2: Partly

**3. Has the statistical analysis been performed appropriately and rigorously?**

Reviewer #1: Yes

Reviewer #2: No

**4. Have the authors made all data underlying the findings in their manuscript fully available?**

**The PLOS Data policy requires authors to make all data underlying the findings described in their manuscript fully available without restriction, with rare exception (please refer to the Data Availability Statement in the manuscript PDF file). The data should be provided as part of the manuscript or its supporting information, or deposited to a public repository. For example, in addition to summary statistics, the data points behind means, medians and variance measures should be available. If there are restrictions on publicly sharing data—e.g. participant privacy or use of data from a third party—those must be specified**.

Reviewer #1: Yes

Reviewer #2: Yes

**5. Is the manuscript presented in an intelligible fashion and written in standard English?**

**PLOS ONE does not copyedit accepted manuscripts, so the language in submitted articles must be clear, correct, and unambiguous. Any typographical or grammatical errors should be corrected at revision, so please note any specific errors here.**

Reviewer #1: No

Reviewer #2: Yes

**6. Review Comments to the Author**

**Please use the space provided to explain your answers to the questions above. You may also include additional comments for the author, including concerns about dual publication, research ethics, or publication ethics. (Please upload your review as an attachment if it exceeds 20,000 characters)**

**Reviewer #1: Methods and Results**

**Please make sure that the temperatures in the methods match the temperatures in the figure legends. As a formatting issue, I’m not sure why the figure legends are incorporated into the results section.**

Sorry to make confusing the figure legends. We fixed the temperature.

Fig 1. Inhibition of *Canddia* spp. biofilm formation by *H. rhombea* extract

The biofilm formation of (A) *C. albicans*, (B) *C. tropicalis var. tropicalis*, (C) *C. parapsilosis var. parapsilosis*, (D) *C. glabrata*, (E) *C. tropicalis*, and (F) *C. parapsilosis* was induced in YPD media supplemented with 10% fatal bovine serum with the indicated concentrations of *H. rhombea* extract at 37 ℃ for 24h.

Fig 2. *H. rhombea* extracts increased the susceptibility of *C. albicans* to miconazole etc.

*H. rhombea* extract (6.25μg/mL) increased the susceptibility of miconazole (3.125μg/mL) (A), magnoflorine (3.125μg/mL) (B) and dioscin (3.125μg/mL) (c) against *C. albicans*. The biofilm formation was induced for 24 h by growing of *C. albicans* in YPD media supplemented with 10% fetal bovine serum. MCZ: Miconazole, MF: Magnoflorine, Do: Dioscin, HR: *H. rhombea* extract.

Fig. 3 Inhibition of *C. albicans* dimorphic transition by *H. rhombea* extract in different hyphal-inducing media.

(A) *C. albicans* dimorphic transition was induced using RPMI 1640 and images of *C. albicans* cells (B) were obtained using a microscope. A: *C. albicans* without extract, B: *C. albicans* with 1.56μg/mL of extract treated, C: *C. albicans* with 6.25μg/mL of extract treated, D: *C. albicans* with 25μg/mL of extract treated.

(C) *C. albicans* dimorphic transition was induced using 10% FBS YPD medium and images of *C. albicans* cells (D) were obtained using a microscope. A: *C. albicans* without extract, B: *C. albicans* with 1.56μg/mL of extract treated, C: *C. albicans* with 6.25μg/mL of extract treated, D: *C. albicans* with 25μg/mL of extract treated.

Fig. 4 *H. rhombea* extract inhibited adhesion of *C. albicans* to HaCaT cells.

*C. albicans* (1 × 10^6^ cells/mL) with indicated concentration of *H. rhombea* extract were incubated at 37°C for 24 h and *Candida* cells that were remaining on the HaCaT cells were counted.

Fig. 5 *H. rhombea* extract inhibited the expression of genes related to biofilm formation and virulence of *C. albicans.*

*H. rhombea* extract reduced the expression of genes related with biofilm formation (A), Ras1-cAMP-Efg1 pathways (B), hyphal-specific (C) and extracellular matrix (D).

Total RNA was extracted from *C. albicans* treated with the indicated concentration of *H. rhombea* extract using RNA extraction kit, converted to cDNA, and analyzed by qPCR with the respective primers.

**I have concerns with the results reported in figure 3 (inhibition of filamentation). The following description is difficult to understand “This method, which was necessary because the hyphae-specific identification cells could not be quantified, also resulted in an under expression of the number of mycelial cells in the population. The reported percentage of hyphae is normalized to the percentage of probability hyphae when a numerator is not added. Since the number of cells observed in D is small, it seems to be the part where the number of observed cells is small.”**

: Thanks for your suggestion. We have modified the experimental method for the problematic part.

*C. albicans* were grown overnight in YPD medium. 1 × 10^6^ cells/mL of *Candida* with or without extracts were incubated in RPMI 1640 medium and YPD media supplemented with 10% fetal bovine serum medium to induce dimorphic transition at 37°C for 4 h. Inhibition quantification of the yeast-to-hyphal-form transition was accomplished by counting the number of hyphae cells in the population as previously described [26, 32, 33, 34, 35, 36, 37, 38, 39, 40]. More than 1,000 cells were counted for each well in duplicate, and all assays were repeated five times. Representative results of images were obtained using a fluorescence microscope (EVOS® FL, ThermoFisher Scientific, Waltham, MA, USA). The experiments were performed in triplicate.

**What does “the percentage of probability hyphae” mean?**

: Thanks for your suggestion. We have modified the experimental method for the problematic part.

*C. albicans* were grown overnight in YPD medium. 1 × 10^6^ cells/mL of *Candida* with or without extracts were incubated in RPMI 1640 medium and YPD media supplemented with 10% fetal bovine serum medium to induce dimorphic transition at 37°C for 4 h. Inhibition quantification of the yeast-to-hyphal-form transition was accomplished by counting the number of hyphae cells in the population as previously described [26, 32, 33, 34, 35, 36, 37, 38, 39, 40]. More than 1,000 cells were counted for each well in duplicate, and all assays were repeated five times. Representative results of images were obtained using a fluorescence microscope (EVOS® FL, ThermoFisher Scientific, Waltham, MA, USA). The experiments were performed in triplicate.

**The table in the supplementatal information indicates that the percentage graphed is the average number from a particular treatment as a percentage of the number in the untreated control. Presumably these numbers are the number of hyphal cells. I don’t see the rationale for expressing this information as a percentage of the control. Surely graphing the percentage of hyphal cells out of the total number of cells counted in a particular treatment would be clearer.**

**Numerous language issues remain. Throughout it should be susceptibility to an antifungal, not susceptibility of an antifungal.**

**Fig. 3**

**(A)**

(C)

**Line 52 should have “pathogenesis” instead of pathogenic.**

: Thanks for your suggestion. Based on your advice, we have changed word.

One of the features of Candida species pathogenesis is their ability to form biofilms, and nosocomial infections are often related to the ability to produce biofilm on mucosal surfaces and implanted medical devices [2, 3, 4, 5].

**Line 57 The following does not make sense, although I have an idea of what the second part is trying to say “The biofilm matrix acts as an intestinal for sessile cells, avert the inlet of the majority used antifungal agents, therefore give drug resistance.”**

: Thank you very much. We made the corrections as you mentioned.

The biofilm matrix highly acts to structure microbial communities include sessile cells that are frequently much more resistant to antifungal agents

**Line 65 should read “Traditional medicine is proposed to have lower adverse reactions compare with typical medicines.”**

: Thanks for your constructive suggestions. We have made corrections in that regard based on your advice.

Herbal medicines usually have fewer side effects compared to over-the-counter medicines.

**Line 92 What does “Controls without test compounds were served” mean?**

: Thank you very much. Based on your advice, we have changed word.

Wells without test compounds served as controls (DMSO concentration of 0.1%).

**Line 95 something like “for 24h, non-adherent cells were removed by washing with PBS and then 100ul of 1% aqueous crystal violet was applied for 30 minutes” might be better**

: Thank you for your suggestion. Based on your advice, we have added the manuscript modified portion shown on line 95.

After incubation at 37°C for 24h, non-adherent cells were removed by washing with PBS and then 100ul of 1% aqueous crystal violet was applied for 30 minutes.

**Line 112 (and others) should be fetal, not fatal.**

: Thank you very much. Based on your advice, we have changed word.

*C. albicans* were grown overnight in YPD medium. 1 × 10^6^ cells/mL of Candida with or without extracts were incubated in RPMI 1640 medium and YPD media supplemented with 10% fetal bovine serum medium to induce dimorphic transition at 37°C for 4 h.

**Line 116: the term bud would be best, not “sprout cell” and this whole description could be simplified.**

: Thanks for your constructive suggestions. We have modified the experimental method for the problematic part.

*C. albicans* were grown overnight in YPD medium. 1 × 10^6^ cells/mL of *Candida* with or without extracts were incubated in RPMI 1640 medium and YPD media supplemented with 10% fetal bovine serum medium to induce dimorphic transition at 37°C for 4 h. Inhibition quantification of the yeast-to-hyphal-form transition was accomplished by counting the number of hyphae cells in the population as previously described [26, 32, 33, 34, 35, 36, 37, 38, 39, 40]. More than 1,000 cells were counted for each well in duplicate, and all assays were repeated five times. Representative results of images were obtained using a fluorescence microscope (EVOS® FL, ThermoFisher Scientific, Waltham, MA, USA). The experiments were performed in triplicate.

**Line 133 should read “non-adherent” for “none adhere”**

: Thank you very much. we have added the manuscript modified portion shown on line 133.

DMEM were drained and then plates were cautiously washed three times with PBS to remove none adhere cell.

**Line 158. This might be better described as a growth inhibition assay, as that is what is being assessed, not killing.**

: Thanks for your suggestion. Modified according to your advice.

Growth inhibition assay for *C. albicans*

**Line 187 should read “Extract was applied”**

: Thank you very much. We have made corrections in that regard based on your advice.

*H. rhombea* extract was applied increased the susceptibility of antifungal agents against *C. albicans.*

**Line 189 should read “increased the susceptibility to miconazole”**

: Thank you for your careful work. We have added changed of the word.

Treatment of 3.125μg/mL of extract increased the susceptibility to miconazole with 99% of fungal growth inhibition from 38% inhibition by miconazole treatment.

**Line 190 I think this line should read “The extract also increased the susceptibility to plant-derived antifungal candidates including”**

: Thank you for your professional advice. According to the guide for author, we carefully revised changed the word.

The extract also increased the susceptibility to plant-derived antifungal candidates including magnoflorine (99% growth inhibition by 6.25μg/mL of extract compared with 29% growth inhibition by 3.125ug/ml of only magnoflorine treatment) and dioscin (99% growth inhibition by 6.25μg/mL of extract compared with 39% growth inhibition by 3.125ug/ml of only dioscin treatment) (Fig 2A, 2B and 2C).

**Fig 2 Legend should read increased the susceptibility of C. albicans to miconazole etc.**

: Thank you for your valuable suggestions. We have modified

Fig 2. *H. rhombea* extract increased the susceptibility of *C. albicans* to miconazole etc.

**Line 205: what is meant by “serious infection effect”?**

: Thank you for your careful work. We have added changed of the word.

The yeast-to-hyphae conversion is an important virulence-mediated property of *C. albicans.* The formation of hyphae aids the subsequent invasive growth of *C. albicans* to penetrate host tissues and lead to the establishment of systemic infection [7]. Extract tested whether the dimorphic transition was influenced and found 1.56μg/mL of the extract significantly inhibited hyphae formation in RPMI 1640 or a 10% FBS YPD medium, and extract with higher than 6.25μg/mL completely blocked the hyphae formation (Fig 3A, 3B, 3C and 3D).

**Line 286: perhaps “further studies must be conducted.”**

: Thank you for your constructive suggestion. As you suggested.

*H. rhombea* extract inhibited *Candida* biofilm formation that suggests there should be a common mechanism to induce the biofilm among the Candida species, but further studies must be conducted.

**Line 293: gene names must be italicized**

: Thanks for your constructive suggestions. As you suggest, I have corrected that part.

*Candida* species biofilm formation is determined by various transcription factors including *BCR1, EFG1, TEC1,* and *NDT80* that function as a component part in several pathways is also participated to the adherence of *Candida* suggested that even though biofilm formation is initially tested, adherence or infection factors could be also regulated by treatment of *H. rhombea* extract.

**Line 294: perhaps “that influence adhesion” in place of “and is also participated to the adherence”**

: Thank you for your careful reading of our manuscript. According to your comments, we have carefully changed this part.

*Candida* species biofilm formation is determined by various transcription factors including *BCR1, EFG1, TEC1,* and *NDT80* that function as a component part in several pathways and is also participated to the adherence of *Candida* suggested that even though biofilm formation is initially tested, adherence or infection factors could be also regulated by treatment of *H. rhombea* extract.

**Line 295 “could” would be better than “should”**

: Thank you for your valuable comments. According to your suggestion.

*Candida* species biofilm formation is determined by various transcription factors including *BCR1, EFG1, TEC1,* and *NDT80* that function as a component part in several pathways is also participated to the adherence of *Candida* suggested that even though biofilm formation is initially tested, adherence or infection factors could be also regulated by treatment of *H. rhombea* extract.

**Line 296 should read “Further studies, including…”**

: Thanks for your constructive suggestion and we have made the modification.

Further studies, including the precise target determination of *H. rhombea* extract, effector components, and in vivo testing must be carried out.

**In figure 5 Planktonic has been misspelled**

: Thank you for your careful review. The part you mentioned has been removed.

**Reviewer #2: This revised manuscript is much improved, I thank the authors for their hard work. I still have some remaining concerns and a few language corrections.**

**My major concern relates to their plant leaf extract and the use of RPMI. Many plant leaf extracts are acidic.**

**RPMI as used in the candida field is typically heavily buffered with MOPS. It is not clear from the description of the methods that RPMI was buffered. Candida filamentation and biofilm formation are heavily influenced by pH. Please clarify the situation with the RPMI.**

: Thank you for your careful reading of our manuscript. We conducted the experiment with reference to these three reference documents.

Yang L, Liu X, Zhuang X, Feng X, Zhong L, Ma T. Antifungal Effects of Saponin Extract from Rhizomes of *Dioscorea panthaica* Prain et Burk against *Candida albicans*. Evid Based Complement Alternat Med 2018; 2018:6095307. doi: 10.1155/2018/6095307 PMID: 29853962

Zhang LL, Lin H, Liu W, Dai B, Yan L, Cao YB, et al. Antifungal Activity of the Ethanol Extract from *Flos Rosae Chinensis* with Activity against Fluconazole-Resistant Clinical *Candida*. Evid Based Complement Alternat Med. 2017; 2017:4780746. doi: 10.1155/2017/4780746 PMID: 28303159

Bonifácio BV, Vila TVM, Masiero IF, Silva PBD, Silva ICD, Lopes ÉDO, et al. Antifungal Activity of a Hydroethanolic Extract From *Astronium urundeuva* Leaves Against *Candida albicans* and *Candida glabrata*. Front Microbiol 2019; 10: 2642. doi: 10.3389/fmicb.2019.02642 PMID: 31803166

**My second concern with the revision is that the discussion still does not address competing (and conflicting data) (https://doi.org/10.4014/jmb.1701.01026 J. Microbiol. Biotechnol. (2017), 27(7), 1242–1248) which showed less influence from Hederea rhombea. This should be referenced and discussed**.

: Thank you very much. We carefully checked the references again to make sure was correct.

In the case of the *H. rhombea* we tested, no inhibition of growth was observed. However, in the experiments in the reference literature [29], it was confirmed that the result of *H. rhombea* inhibiting growth. The reason might be the differences in the method of extracting plants.

**The controls still aren't clear. Presumably these are just DMSO. But this needs to be clearly stated when it is used. There is a comment that "controls without test compounds were served" but it should clearly state somewhere is the 0/control used is DMSO**

: Thanks for your careful work. As you mentioned, I have edited and written that part.

Wells without test compounds served as controls (DMSO concentration of 0.1%).

**The title is misleading and should really read something like "Hedera rhombea inhibits the biofilm formation of Candida and increases the susceptibility to antifungal agents"**

**Below are minor language changes to improve the manuscript**.

: Thank you so much. We have checked the whole manuscript carefully according to your advice to improve the readability and accuracy.

*Hedera rhombea* inhibits the biofilm formation of *Candida* and increases the susceptibility to antifungal agents

**Abstract/Page 8**

**line33 should read "Treatment with 6.25....." and "...increased susceptibility to miconazole..."**

: Thank you for your constructive suggestions. According to the guide for author, we carefully revised the word.

Treatment with 6.25μg/mL of extract increased susceptibility to miconazole from 32% decreasing of fungal growth to 98.8% of that based on the fungal growth assay.

**line39 should read "...analysis was done and the extract was found to dose dependently reduce the expression....**

: Thank you very much. We have checked and corrected the points you mentioned.

To know the molecular mechanisms of biofilm formation inhibition by extract, qRT-PCR analysis was done, and the extract was found to dose dependently reduce the expression of hyphal-associated genes (*ALS3, ECE1, HWP1, PGA50*, and *PBR1*), extracellular matrix genes (*GSC1, ZAP1, ADH5,* and *CSH1*), Ras1-cAMP-PKA pathway genes (*CYR1, EFG1*, and *RAS1*), Cph2-Tec1 pathway gene (*TEC1*) and MAP kinases pathway gene (*HST7*).

**Page 9**

**line 44 should read " biofilm formation is good screen for developing.....**

: Thank you for pointing this out. We have corrected the word you pointed out.

In this study, *Hedera rhombea* extract showed inhibition of fungal biofilm formation, activation of antifungal susceptibility, and reduction of infection. These results suggest that fungal biofilm formation is good screen for developing the antifungal adjuvant and *Hedera rhombea* extract should be a good candidate against biofilm-related fungal infection.

**Introduction Page 10**

**line 50 should read "....persist inside the host and can be aided by drug resistance traits which often lead to failure of....."**

: Thank you for your constructive suggestion. As you suggested, we changed word.

*C. albicans* can persist inside the host and can be aided by drug resistance traits which often lead to failure of therapeutic strategies [1].

**line 52 should read "...species pathogenicity is their..."**

: Thank you for your constructive suggestion. We have edited that part.

**line 56 should read "...is a finely controlled process that involves attachment to surface and embedment in the exopolymer...."**

**:** Thank you very much. We have made the modification.

The formation of biofilms involves multiple interconnected signaling pathways [6, 7, 8, 9, 10, 11, 12, 13, 14], and is a finely controlled process that involves attachment to surface and embedment in the exopolymer extracellular matrix [15, 16, 17, 18].

**line 57 should read "the biofilm matrix acts as a haven for sessile cells..."**

**:** Thank you for your constructive suggestion. We have edited that part.

**line 58 should read "....averting the inlet of the majority of anitfungal agents, thereby resulting in resistance"**

: Thank you for your constructive suggestion. We have edited that part.

**line 59 I think the authors mean to say "adherent C. albicans cells without specific drug resistant gene expression..."**

**:** Thank you for your valuable suggestions. We have modified

In fact, adherent *C. albicans* cells without specific drug resistant gene expression are up to 1,000 times more resistant to common antifungal agents than planktonic cells [19].

**line 67 should read "...provenance of replacement remedies...."**

: Thanks for your suggestion. Modified according to your advice.

Accordingly, medicinal plants should be a new provenance of replacement remedies to treat *Candida* infectious diseases [24, 25, 26].

**line 73 should read "Interestingly, the activity of the extract also increased susceptibility to antibiotics."**

: Thank you very much. We made the corrections as you mentioned.

Interestingly, the activity of the extract also increased susceptibility to antibiotics and reducing fungal infection.

**Methods**

**line 92 should probably read "DMSO controls without test compounds were used"**

: Thank you for your valuable comments. According to your suggestion.

DMSO controls without test compounds were used.

**line 93 ...was this RPMI MOPS buffered????**

: Thank you for your careful reading of our manuscript. We conducted the experiment with reference to these three reference documents.

Yang L, Liu X, Zhuang X, Feng X, Zhong L, Ma T. Antifungal Effects of Saponin Extract from Rhizomes of *Dioscorea panthaica* Prain et Burk against *Candida albicans*. Evid Based Complement Alternat Med 2018; 2018:6095307. doi: 10.1155/2018/6095307 PMID: 29853962

Zhang LL, Lin H, Liu W, Dai B, Yan L, Cao YB, et al. Antifungal Activity of the Ethanol Extract from *Flos Rosae Chinensis* with Activity against Fluconazole-Resistant Clinical *Candida*. Evid Based Complement Alternat Med. 2017; 2017:4780746. doi: 10.1155/2017/4780746 PMID: 28303159

Bonifácio BV, Vila TVM, Masiero IF, Silva PBD, Silva ICD, Lopes ÉDO, et al. Antifungal Activity of a Hydroethanolic Extract From *Astronium urundeuva* Leaves Against *Candida albicans* and *Candida glabrata*. Front Microbiol 2019; 10: 2642. doi: 10.3389/fmicb.2019.02642 PMID: 31803166

**line 112... I think autocorrect likely altered this as fatal bovine serum is back/still there. It should be fetal or foetal. This error recurs...see line 130**

**:** Thank you for pointing this out. We have corrected the word you pointed out.

*C. albicans* were grown overnight in YPD medium. 1 × 10^6^ cells/mL of Candida with or without extracts were incubated in RPMI 1640 medium and YPD media supplemented with 10% fetal bovine serum medium to induce dimorphic transition at 37°C for 4 h.

A human epithelial keratinocyte HaCaT cells were maintained in DMEM supplemented with 10% fetal bovine serum at 37°C in a humidified atmosphere of 5% CO_2_.

**Results**

**line 185 should read "H. rhombea extract increased the efficacy of antifungal agents...."**

**If you want to use susceptibility then the extract would increase the susceptibility of C. albicans to antifungals.**

**:** Thank you for your careful reading of our manuscript. we have carefully changed this part.

**Discussion**

**line 285-286. should read "....but further studies should be undertaken."**

: Thank you for your valuable suggestions. We have modified the word.

Moreover, *H. rhombea* increased the antifungal activity of miconazole, magnoflorine, and dioscin (Fig 2), and reduced *Candida* infection (Fig 3 and 4), these results confirmed that biofilm formation is related to the susceptibility of antifungal agents and fungal infection, but further studies should be undertaken.
