# Supplementary material for: Hedera rhombea inhibits the biofilm formation of Candida, thereby increases the susceptibility to antifungal agent, and reduces infection
Source: PLoS One. 2021 Oct 6;16(10):e0258108. doi: 10.1371/journal.pone.0258108 (PMC8494327; doi:10.1371/journal.pone.0258108)
Supplement: Attachment — Submitted filename: Rebuttal 09.16.docx [file pone.0258108.s004.docx]

*Hedera rhombea* inhibits the biofilm formation of *Candida*, thereby increases the susceptibility to antifungal agent, and reduces infection.

We thank the editors and the reviewers for their thoughtful and helpful comments. We have addressed, in a point-by-point manner, all the suggestions and queries from the journal and the reviewer and marked with red in manuscript. The input from the reviewers has allowed us to improve the clarity and quality of our paper. We have included below our point-by-point response to the reviewers’ comments and have included these additions and alterations to the revised manuscript.

Reviewers' comments:

Reviewer's Responses to Questions

Comments to the Author

1. If the authors have adequately addressed your comments raised in a previous round of review and you feel that this manuscript is now acceptable for publication, you may indicate that here to bypass the “Comments to the Author” section, enter your conflict-of-interest statement in the “Confidential to Editor” section, and submit your "Accept" recommendation.

Reviewer #1: (No Response)

Reviewer #2: (No Response)

2. Is the manuscript technically sound, and do the data support the conclusions?

The manuscript must describe a technically sound piece of scientific research with data that supports the conclusions. Experiments must have been conducted rigorously, with appropriate controls, replication, and sample sizes. The conclusions must be drawn appropriately based on the data presented.

Reviewer #1: Yes

Reviewer #2: Yes

3. Has the statistical analysis been performed appropriately and rigorously?

Reviewer #1: Yes

Reviewer #2: Yes

4. Have the authors made all data underlying the findings in their manuscript fully available?

The PLOS Data policy requires authors to make all data underlying the findings described in their manuscript fully available without restriction, with rare exception (please refer to the Data Availability Statement in the manuscript PDF file). The data should be provided as part of the manuscript or its supporting information, or deposited to a public repository. For example, in addition to summary statistics, the data points behind means, medians and variance measures should be available. If there are restrictions on publicly sharing data—e.g. participant privacy or use of data from a third party—those must be specified.

Reviewer #1: Yes

Reviewer #2: Yes

5. Is the manuscript presented in an intelligible fashion and written in standard English?

PLOS ONE does not copyedit accepted manuscripts, so the language in submitted articles must be clear, correct, and unambiguous. Any typographical or grammatical errors should be corrected at revision, so please note any specific errors here.

Reviewer #1: Yes

Reviewer #2: Yes

6. Review Comments to the Author

Please use the space provided to explain your answers to the questions above. You may also include additional comments for the author, including concerns about dual publication, research ethics, or publication ethics. (Please upload your review as an attachment if it exceeds 20,000 characters)

Reviewer #1: I appreciate the changes made by the authors and I have only a handful of minor edits remaining.

Line 57 should read “The biofilm matrix acts to structure microbial communities and includes sessile cells that are frequently much more resistant to antifungal agents.”

: Thanks for your suggestion. We have modified the introduction of the problematic part.

The formation of biofilms involves multiple interconnected signaling pathways [6, 7, 8, 9, 10, 11, 12, 13, 14], and is a finely controlled process that involves attachment to surface and embedment in the exopolymer extracellular matrix [15, 16, 17, 18]. The biofilm matrix acts to structure microbial communities and includes sessile cells that are frequently much more resistant to antifungal agents. In fact, adherent *C. albicans* cells without specific drug resistant gene expression are up to 1,000 times more resistant to common antifungal agents than planktonic cells [19]. Therefore, the biofilm of *C. albicans* is a reservoir of viable fungal cells that can potentially cause systemic infections, with a mortality rate of around 40-60% [20, 21].

Line 74 I think “and reducing fungal infection” needs to be removed since virulence wasn’t assessed in the manuscript.

: Thanks for your suggestion. Based on your advice, we have changed word.

In this study, *H. rhombea* extract showed anti-biofilm formation activity against several fungi including *C. albicans, C. tropicalis, C. glabrata*, and *C. parapsilosis*. Interestingly, the activity of the extract also increased susceptibility to antibiotics. These results suggest that *H. rhombea* extract can be used as a potential anti-fungal adjuvant to control the biofilm-related infection.

Line 125 should read “nonadherent”

: Thanks for your constructive suggestions. We have made corrections in that regard based on your advice.

A human epithelial keratinocyte HaCaT cells were maintained in DMEM supplemented with 10% fetal bovine serum at 37°C in a humidified atmosphere of 5% CO_2_.

HaCaT cells (0.5×10^6^ cells per wall) were grown to confluence on 24 well plates for 24 h. DMEM were drained and then plates were cautiously washed three times with PBS to remove nonadherent cell.

Line 179 remove “was applied”

: Thank you very much. Based on your advice, we have changed word.

*H. rhombea* extract increased the susceptibility of antifungal agents against *C. albicans.*

Line 196 should just read “virulence” not “virulence-mediated”

: Thanks for your suggestion. Modified according to your advice.

The yeast-to-hyphae conversion is an important virulence property of *C. albicans*.

Line 290 I think this should read ”Candida albicans biofilm formation is determined by various transcription factors including BCR1, EFG1, TEC1, and NDT80 that function as components in several pathways and also influence adherence of Candida, suggesting that even though biofilm formation was initially tested, adherence or infection factors could be also regulated by treatment of H. rhombea extract.”

: Thank you very much. We have made corrections in that regard based on your advice.

*C. albicans* biofilm formation is determined by various transcription factors including *BCR1, EFG1, TEC1, and NDT80* that function as components in several pathways and influence adherence of *Candida*, suggesting that even though biofilm formation was initially tested, adherence or infection factors could be also regulated by treatment of *H. rhombea* extract.

Figure 1 Legend has one last “fatal” instead of “fetal”

: Thank you for your valuable suggestions. We have modified

The biofilm formation of (A) *C. albicans*, (B) *C. tropicalis var. tropicalis*, (C) *C. parapsilosis var. parapsilosis*, (D) *C. glabrata*, (E) *C. tropicalis*, and (F) *C. parapsilosis* was induced in YPD media supplemented with 10% fetal bovine serum with the indicated concentrations of *H. rhombea* extract at 37 ℃ for 24h.

Figure 5 be sure that in the figure the label is “planktonic”.

: Thank you for your careful review. The part you mentioned has been removed.

Reviewer #2: This revised manuscript is much improved, I thank the authors for their

hard work. My main remaining concern and is with regards to the RPMI based experiments.

The authors provided references to respond to this question. The frontiers paper that was referenced clearly describes the RPMI they used as supplemented with L-glutamine and buffered with MOPS to pH 7. (see extract 1 below..copied from the article).

The other two papers reference the CLSI standard and then they describe RPMI without sodium barcarbonate at pH7. The CLSI standard recommends the use of RPMI without sodium bicarbonate and with glutamine...but it also says to use a buffer and recommends mops (see second extract below).

If this is accurate for the experiments described here then a similar line should be added to methods. If not perhaps the authors could include a line about the pH of their extract. Or comment on the color of the media as the phenol red is in RPMI as a pH indicator.

Alternatively RPMI can use a sodium bicarbonate/Co2 buffer system....but that is usually for cell culture.

[extract 1] taken from Front. Microbiol., 15 November 2019 | https://doi.org/10.3389/fmicb.2019.02642 "A round-bottom 96-well microplate (Corning Inc., Corning, NY, United States) was filled with RPMI medium supplemented with L-glutamine (Cellgro; Corning, United States) and buffered with 165 mM morpholinepropanesulfonic acid (MOPS) (Thermo Fisher Scientific Inc., United States) at pH 7.0 "

[extract 2] taken from CLSI M27 standard for antifungal testing: "Media should be buffered to a pH of 7.0 ± 0.1 at 25 °C. A buffer should be selected that does not antagonize antifungal agents. Tris buffer is unsatisfactory, because it antagonizes the activity of flucytosine. Zwitterion buffers are preferable to buffers that readily traverse the cell membrane, such as phosphate buffers, because, theoretically, the latter can produce unexpected interactions with antifungal agents. One buffer that has been found to be satisfactory for antifungal testing is MOPS [3-(N-morpholino) propanesulfonic acid] (final concentration 0.165 mol/L for pH 7.0). The pH of each batch of medium is to be checked with a pH meter immediately after the medium is prepared; the pH should be between 6.9 and 7.1 at room temperature (25 °C). MIC performance characteristics of each batch of broth are evaluated using a standard set of quality control organisms."

: Thank you for your valuable suggestions. We have modified

*C. albicans* were grown overnight in YPD medium. 1 × 10^6^ cells/mL of *Candida* with or without extracts were incubated in RPMI 1640 medium and YPD media supplemented with 10% fetal bovine serum medium to induce dimorphic transition at 37°C for 4 h. RPMI without sodium barcarbonate and with glutamine buffered with MOPS [3-(N-morpholino) propanesulfonic acid] to pH 7. Inhibition quantification of the yeast-to-hyphal-form transition was accomplished by counting the number of hyphae cells in the population as previously described [26, 32, 33, 34, 35, 36, 37, 38, 39, 40]. More than 1,000 cells were counted for each well in duplicate, and all assays were repeated five times. Representative results of images were obtained using a fluorescence microscope (EVOS® FL, ThermoFisher Scientific, Waltham, MA, USA). The experiments were performed in triplicate.
